# Unleashing the immune response to NY-ESO-1 cancer testis antigen as a potential target for cancer immunotherapy

**DOI:** 10.1186/s12967-020-02306-y

**Published:** 2020-03-27

**Authors:** Afsheen Raza, Maysaloun Merhi, Varghese Philipose Inchakalody, Roopesh Krishnankutty, Allan Relecom, Shahab Uddin, Said Dermime

**Affiliations:** 1 National Center for Cancer Care and Research, Hamad Medical Corporation, Doha, Qatar; 2 Translational Cancer Research Facility and Clinical Trial Unit, Interim Translational Research Institute, Hamad Medical Corporation, Doha, Qatar; 3 Translational Research Institute, Hamad Medical Corporation, Doha, Qatar; 4 Hamad Medical Corporation, iTRI, Hamad Medical City (Building 320, Office 3-6-5), Po Box 3050, Doha, Qatar

**Keywords:** Cancer immunotherapy, Cancer testis antigen, Cancer vaccine, Immune checkpoint inhibitors, NY-ESO-1, Tumor microenvironment

## Abstract

**Introduction:**

Cancer Immunotherapy has recently emerged as a promising and effective modality to treat different malignancies. Antigenic profiling of cancer tissues and determination of any pre-existing immune responses to cancer antigens may help predict responses to immune intervention in cancer. NY-ESO-1, a cancer testis antigen is the most immunogenic antigen to date. The promise of NY-ESO-1 as a candidate for specific immune recognition of cancer comes from its restricted expression in normal adult tissue but frequent occurrence in multiple tumors including melanoma and carcinomas of lung, esophageal, liver, gastric, prostrate, ovarian, and bladder.

**Main body:**

This review summarizes current knowledge of NY-ESO-1 as efficient biomarker and target of immunotherapy. It also addresses limitations and challenges preventing a robust immune response to NY-ESO-1 expressing cancers, and describes pre-clinical and clinical observations relevant to NY-ESO-1 immunity, holding potential therapeutic relevance for cancer treatment.

**Conclusion:**

NY-ESO-1 induces strong immune responses in cancer patients but has limited objective clinical responses to NY-ESO-1 expressing tumors due to effect of competitive negative signaling from immune-checkpoints and immune-suppressive tumor microenvironment. We propose that combination therapy to increase the efficacy of NY-ESO-1 specific immunotherapeutic interventions should be explored to unleash the immune response against NY-ESO-1 expressing tumors.

## Introduction

Carcinogenesis may often lead to the expression of neo-antigens recognized, under certain conditions, by the immune system [1]. However, expression of tumor-associated antigens (TAA) does not commonly lead to effective tumor cell eradication due to inconsistent expression, immunological tolerance, lack of restriction to transformed tissues, low affinity of the TCR to the MHC/peptide complex or immunosuppressive microenvironment [2]. Different from most TAA, cancer testis antigens (CTA) are considered highly immunogenic due to their unique set of characteristics including restricted expression in immune privileged organs, stable expression on tumor tissues and ability to encode immunogenic antigens to cancer [3]. To date, an estimated 250 proteins associated with CTA group have been documented [4]. Of these, NY-ESO-1 is a particularly promising target for immunotherapy due to its high and frequent expression in malignancies and its ability to elicit potent integrated natural humoral and cellular responses [5]. This has led to a number of pre-clinical studies and clinical trials (completed and ongoing) exploring the potential efficacy of immunotherapeutic strategies against NY-ESO-1 expressing tumors [6]. Here, we aim to provide an in-depth perspective on NY-ESO-1 as an efficient target for immunotherapy and describe its immunogenicity limitation and challenges that need to be addressed to unleash a robust immune response against cancers. Furthermore, this review will provide detailed perspectives on pre-clinical and clinical advances with relation to NY-ESO-1 that may have therapeutic potential in combination with standard therapies.

### Structure, expression and regulation of NY-ESO-1

**Fig. 1:**
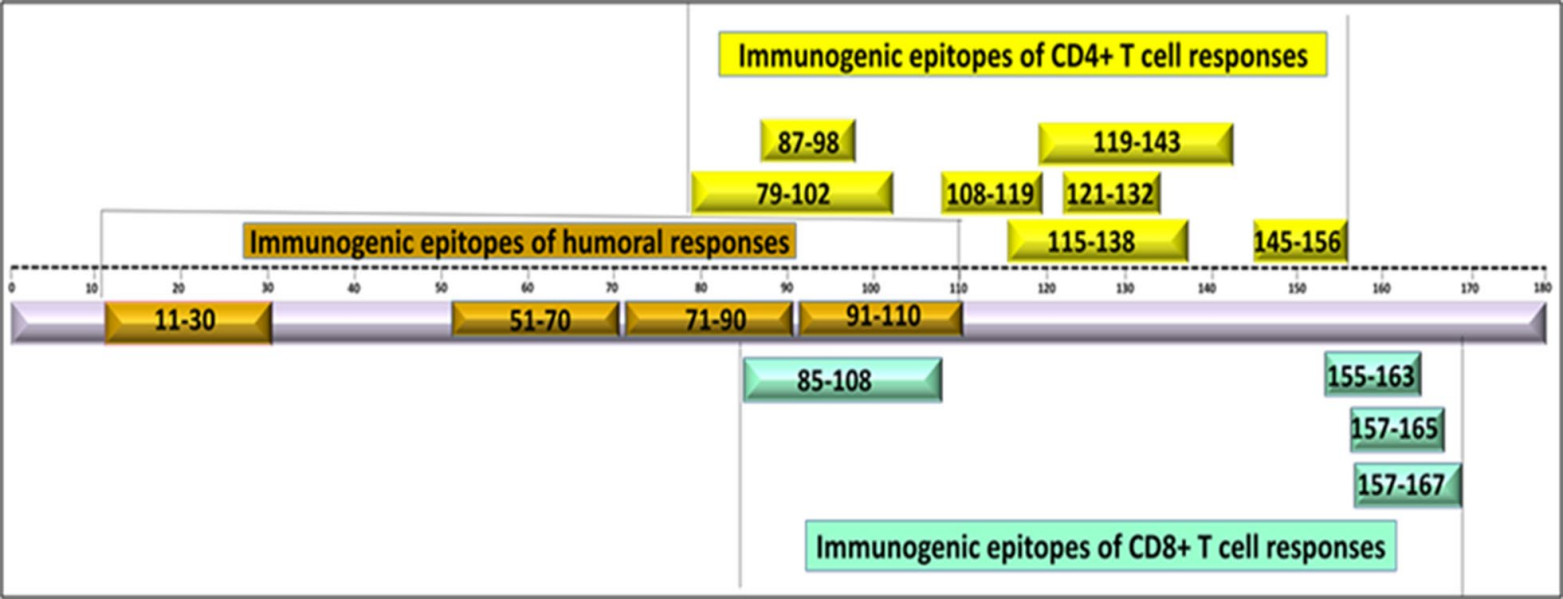
Schematic representation of amino acids representing immunogenic epitopes against anti-NY-ESO-1 antibody, CD4+ and CD8+ T cell responses. Naturally occurring anti-NY-ESO-1 antibodies are mostly mapping to soluble N-terminus region while the cellular responses are mapping to the C-terminus region [139]

NY-ESO-1 belongs to Cancer Testis 6 antigen (CT6) family encoded by CTAG1 gene. Its gene maps to the Xq28 region of the X chromosome. Structurally, it is 180 amino acid long protein of 18 kDa, containing epitopes of humoral and cellular responses in its glycine rich N terminal region and an extremely hydrophobic C terminal region [7–9] (Fig. 1).

In healthy individuals, the expression of NY-ESO-1 appears early at fetal level with germ cells of testis and ovaries expressing this antigen at 13–18 weeks, plateauing at 22–24 weeks and then decreasing rapidly [10]. Therefore, NY-ESO-1 is only expressed in immune privileged organs such as testis and placenta (in spermatogonia/oogonia). Since, testis/placenta do not express MHC alleles, NY-ESO-1 expression is lost during spermatid differentiation. Therefore, it is not expressed in normal healthy tissues. This is an important parameter as any therapeutic potential against NY-ESO-1 will not be restricted by implications of normal tissue damage [5].

In cancers, NY-ESO-1 expression is a consequence of epigenetic event that involves tightly controlled recruitment and sequential interaction of histone deacetylases, histone methyltransferase, DNA methyltransferases, and transcription factors. Mechanistically, formation of multi-protein complexes between HDAC1-mSin3A-NCOR1, Dnmt3b-HDAC1-Egr1and Dnmt1-PCNA-UHRF1-G9a have been reported as main players of NY-ESO-1 regulation [11].

### Factors affecting NY-ESO-1 expression

The expression of NY-ESO-1 in cancers is a well-documented phenomenon with studies reporting that approximately 75% of cancer patients express this antigen at some stage during the course of their illness [12]. However, since tumor characteristics evolve during the course of the disease, it is important to understand that factors such as tumor stage, grade and therapeutic interventions are critical factors directing the expression of NY-ESO-1 in tumors [5, 13, 14]. For e.g. with respect to grade, NY-ESO-1 expression can vary from 0% in grade 1 to 44% in grade 4. Similarly, stage wise, variable expression of NY-ESO-1 has been reported in premalignant lesions, early stage and metastatic tumors [15, 16]. Global data indicates that in majority of tumors, NY-ESO-1 is frequently expressed in metastatic, high grade/advanced stage tumors and is as such associated with poor prognosis [17–21].

In addition to this, studies have also observed relationship between clinical course of disease and NY-ESO-1 expression. For e.g. patients with disease remission show variable patterns with some patients exhibiting antigen loss while others showing stable expression over time [12]. It is postulated that this variable expression pattern may be a result of interrelated tumor-immune dynamics including intra-tumoral heterogeneity, immuno-editing, or reduced tumor cell proliferation [5]. On the other hand, there are reports that therapeutic interventions such as radiotherapy or de-methylating agents enhance the release of NY-ESO-1 antigen from the tumor, and this in itself may play a critical role in directing tumor dynamics [22, 23].

Other factors such as culturing and detection methods are associated with its expression analysis and reporting. For e.g. NY-ESO-1 expression is usually higher in cell lines due to well-controlled homogenous culture conditions [24, 25]. Similarly, detection methods are an important parameter as this has direct effect on reporting of NY-ESO-1 expression. For e.g. reverse transcriptase PCR (RT PCR) and tissue microarray have been reported to give higher false positive/negative results due to variation in the homology/binding affinity of detection antibodies. Immunohistochemistry (IHC), on the other hand is considered a gold standard as its allows localized detection of NY-ESO-1 antigen in the cytoplasm of the tumor tissues [14]. Although, NY-ESO-1 antigen is always localized in the cytoplasm, some limited studies have also reported simultaneous expression of NY-ESO-1 in both nucleus and cytoplasm of tumor tissues. It is unclear if this simultaneous expression, observed in certain cancers, is a stable trait or varies over time [26–28].

### Immunogenicity of NY-ESO-1

#### NY-ESO-1 humoral responses

The discovery of NY-ESO-1 was based on its capacity to induce detectable antibody response in cancer patients. Since its discovery, a large number of studies have documented aberrant expression of NY-ESO-1 in variable frequencies ranging from 10 to 50% among solid tumors, 25–50% of melanomas, and up to 80% in synovial sarcomas [7, 26–36]. A large scale serological study by *Oshima* et al. on 1969 specimens from patients with different cancer types reported highest frequency in esophageal cancer (32%), followed by lung cancer (13%), hepatocellular cancer (11%), prostate and gastric cancer (10%), colorectal cancer (8%) and breast cancer (7%) indicting the variable pattern of antibody response in different cancer types [37].

The predictive/prognostic utility of NY-ESO-1 antibody has been investigated by correlating the antibody levels with patient responses. Valuable observations with respect to antibody responses and tumor burden have been reported. Various studies have reported a pattern of antibody increase with disease progression that decreases with effective treatment [38]. For e.g. a study focusing on NY-ESO-1 antibody in 363 gastric cancers patients showed that NY-ESO-1 antibody was detected in 3.4% (6/176) of stage I, 4.4% (2/45) of stage II, 25.3% (17/67) of stage III and 20.0% (16/75) of stage IV gastric cancer patients, resulting in an overall detection rate of 11.1% (41 of 363). Interestingly, the study observed that patients who underwent surgery and did not suffer a subsequent relapse displayed consistent decreases or complete disappearance of NY-ESO-1 antibody from their sera [39]. Similarly, a study on 155 CRC patients (stage III or IV) reported that out of 24.5% of NY-ESO-1 antibody positive patients, 59 patients exhibited sera conversion after change in their clinical status. This is important evidence indicating correlation between clinical status and NY-ESO-1 humoral response [40].

Another study reported that out of 689 ovarian cancer patients tested, 19.0% that tested positive for NY-ESO-1 antibody exhibited higher stage/grade at presentation with more serous histology. These patients were found to have fewer complete responses to primary therapy with worse outcomes. Interestingly, the study observed that NY-ESO-1 positive patients on antigen-specific immunotherapy exhibited improved response and overall survival indicating that immune dynamics in NY-ESO-1 patients is modulated towards a better clinical trajectory using NY-ESO-1 specific targeted therapy [41].

Studies investigating the utility of NY-ESO-1 antibody as surrogate marker of response in cancers such as multiple myeloma, melanoma, gastric cancer, hepatocellular carcinoma, bladder, prostate cancer etc. have also been performed with promising results [35, 38, 39, 42–44]. For e.g. in synovial sarcoma, strong NY-ESO-1 expression is observed while in spindle cell neoplasms, NY-ESO-1 expression is rare. It is suggested that this distinct expression profile can help to distinguish these two types of sarcomas diagnostically [45]. Similarly, studies have suggested that NY-ESO-1 expression can serve as a sensitive and specific diagnostic biomarker in myxoid and round cell liposarcoma [46–48].

However, it should be noted that circulating antibodies against NY-ESO-1 cannot mediate direct anti-tumor responses. Instead, these antibodies facilitate the formation of immune complexes, with NY-ESO-1 protein, for effective cross presentation by dendritic cells [44]. It is well understood that, in NY-ESO-1 expressing tumors, key anti-tumor responses involve integrated antibody, CD4^+^ and CD8^+^ T cell responses leading to robust immune response with significant clinical benefit [49–52]. Interestingly, clinical trials have shown that, therapeutic interventions against NY-ESO-1(for e.g. vaccination) are capable of robust immune response and tumor control as compared to naturally occurring responses [53, 54]. This is an important understanding as it provides evidence that therapeutic boosting of humoral and cellular responses is a key control for NY-ESO-1 expressing tumors.

#### NY-ESO-1 cellular responses

Structurally, the epitopes for cellular response in NY-ESO-1 are clustered within its central (80–110 aa) and C terminal region (157–170 aa) [8, 9, 55] (Fig. 1). These epitopes are considered highly immunogenic with capability of eliciting potent CD4^+^ and CD8^+^ T cell responses [54]. A number of clinical trials have evidenced on the role of NY-ESO-1 cellular responses in driving therapeutic benefits in patients. For e.g. immunization in patients with NY-ESO-1 specific peptides has shown to induce potent CD8^+^ T cell responses leading to regression and disease stabilization in such patients [51, 56–58]. Another study on full length NY-ESO-1 protein vaccine showed induction of integrated humoral and cellular responses with clinical benefit and overall/progression free survival [55].

#### Factors limiting immunogenic potential of NY-ESO-1

Although, a highly promising therapeutic antigen, there are several factors that limit the induction of efficient responses against NY-ESO-1 expressing tumors including (a) tumor evasion from immune recognition, (b) inefficient induction of high affinity adaptive immunity and (c) tumor induced immunosuppression [59–63]. A number of studies have documented these factors in limiting control of NY-ESO-1 expressing tumors. For e.g. *Lotta von Boehmer* et al. evidenced this in a case of NY-ESO-1 expressing melanoma patient who was started on anti-NY-ESO-1 specific vaccines for tumor control. Though immune responses were boosted initially in the patient, some lesions continued to grow. On the other hand, with lesions still progressing, the expression of NY-ESO-1 on tumor cells was found to be lost with reduced specific CD8+ T cell response. Subsequent immunizations were unable to boost or recall these specific cellular responses. The study postulated that NY-ESO-1-specific immunological pressure/persistence of NY-ESO-1 negative cells lead to tumor evasion and ultimately tumor progression [60]. This complex relationship between NY-ESO-1 expressing tumors and the immune system has been demonstrated in other studies as well [64]. For e.g. in murine xenograft models of multiple myeloma, adoptive transfer of NY-ESO-1_157-165_/HLA-A*02:01-specific T cells was performed. Despite encouraging results for four mice, disease persistence was observed in two of the mice. The study reported a selective loss of HLA-A*02:01 expression indicating that loss of heterozygosity in MHC was the factor leading to immune escape against NY-ESO-1 specific intervention [65]. Similarly, biopsy analysis of 17 melanoma patients, who had relapsed after NY-ESO-1 protein vaccination showed that 11/17 patients exhibited either NY-ESO-1 or HLA class I downregulation, indicating that alteration in tumor phenotype can lead to immune evasion and relapse [59].

On the other hand, several intrinsic and extrinsic immune suppressive mechanisms are reported to be involved in modulation of NY-ESO-1 specific immune responses. These include activation of immune checkpoints such as PD-1, CTLA-4 etc. and infiltration of suppressive cells such as regulatory T cells (Tregs), myeloid-derived suppressor cells (MDSC), various cytokines and chemokines [65–68]. Many studies have documented the restrictive abilities of these cells in CD4^+^ T cell activation and CD8^+^ priming thus limiting cellular responses against NY-ESO-1 [69–72]. For e.g. NY-ESO-1 positive patients with high frequencies of circulating CD25^+ high^/FOXP3^+^ Tregs and CD14^+^/CD11b^+^/HLA-DR^−/low^ MDSCs exhibited poor response to therapeutic intervention. On the other hand, patients with low MDSCs and higher tumor infiltrating lymphocytes (TIL), were showed improved prognosis [71, 73, 74]. Similarly, Tregs depletion by anti-CD25 antibodies has been shown to enhance anti-tumor responses in murine models [75, 76]. Furthermore, therapeutic interventions targeting checkpoint molecules (for e.g anti-PD-1, anti-CTLA-4) work by blocking negative pathways and enhancing anti-NY-ESO-1 specific immunity [67, 77–81].

Therefore, It is clear that various factors influence and direct NY-ESO-1 mediated responses. Thus, immunotherapeutic interventions keeping these factors in perspective could facilitate potentiation of anti-NY-ESO-1 immunogenicity.

#### Current pre-clinical and clinical trials of NY-ESO-1 antigen

The potential of NY-ESO-1 antigen to generate integrated humoral and cellular responses have paved the way for various pre-clinical studies and clinical trials. Most studies are focused on generation of NY-ESO-1-specific TCR gene transduced T lymphocytes, humanized engineered antibodies and combination of NY-ESO-1 petides/proteins with biological agents such as oncolytic viruses and antibody drug conjugate natural dendritic cells, gene modified viruses/bacterial vectors etc. (Tables 1, 2) [82].

**Table 1:** Completed clinical trials targeting New York esophageal squamous cell carcinoma-1(NY-ESO-1) antigen

Immunotherapeutic strategy	Adjuvant/interventions	Indications	Phase	Trial ID
NY-ESO-1 protein vaccine	CpG 7909	Advanced prostate cancer	1	NCT00292045
	Cholesteryl pullulan(CHP) + CHP HER2 + OK-432	Esophageal cancer, lung cancer, stomach cancer, breast cancer, ovarian cancer	1	NCT00291473
	Glucopyranosyl lipid adjuvant stable emulsion (GLA-SE)	Melanoma, ovarian cancer, sarcoma non-small cell lung cancer, breast cancer	1	NCT02015416
	Recombinant Fowl-Pox virus vector	Fallopian tube cancer, ovarian cancer, peritoneal cavity cancer	2	NCT00112957
	Recombinant canarypox virus vector (ALVAC) 2 + TRIad of Co-stimulatory molecules B7-1, ICAM- and LFA-3.	Fallopian tube cancer, ovarian cancer, peritoneal cavity cancer	1	NCT00803569
	ISCOMATRIX	Melanoma	2	NCT00518206
	Imiquimod	Malignant melanoma	1	NCT00142454
NY-ESO-1 peptide vaccine	Montanide, montanide + carboxymethylcellulose, polyinosinic-polycytidylic acid, and poly-l-lysine (Poly-ICLC)	Epithelial ovarian cancer, fallopian tube cancer, primary peritoneal cancer	1	NCT00616941
	CpG 7909 + montanide ISA-51	Stage III/IV;resected Stage II, III, or IV non-small cell lung cancer or esophageal cancer	1	NCT00199836
	None	Prostate cancer	1	NCT00616291
	CpG 7909, CpG 7909 + montanide ISA 720	All NY-ESO-1 expressing tumors	1	NCT00819806
	BCG vaccine, sargramostim	Bladder cancer	1	NCT00070070
	Resiquimod	Melanoma	1	NCT00470379
NY-ESO-1 TCR	Palliative radiation therapy	Sarcoma	1	NCT02319824
NY-ESO-1 specific monoclonal antibody	Resiquimod and/or carboxymethylcellulose, polyinosinic-polycytidylic acid, and poly-l-lysine (poly-ICLC)	Advanced malignancies	1 and 2	NCT00948961

**Table 2:** On-going clinical trials targeting New York esophageal squamous cell carcinoma-1 (NY-ESO-1) antigen

Immunotherapeutic strategy	Adjuvant/interventions	Indications	Phase	Trial ID
Anti-NY-ESO-1 TCR	Cyclophosphamide	Synovial sarcoma, melanoma, esophageal cancer, ovarian cancer, lung cancer, bladder cancer, liver cancer	1	NCT02869217
	Cyclophosphamide + transforming growth factor-beta receptor II (TGFbDNRII)-transduced autologous tumor infiltrating lymphocytes	Adult solid neoplasm	1 and 2	NCT02650986
	Cyclophosphamide	Bone Sarcoma, soft tissue sarcoma, melanoma, liver cancer, esophageal cancer, breast cancer, thyroid cancer, ovarian cancer	1	NCT03159585
	None	Advanced malignant solid tumors	1	NCT03047811
	Cyclophosphamide, fludarabine	Bladder carcinoma, breast cancer, esophagus carcinoma, lung cancer, melanoma, multiple myeloma, neuroblastoma, ovarian cancer, synovial sarcoma, other metastatic solid cancers	1	NCT02457650
	Aldesleukin, cyclophosphamide, decitabine	Recurrent fallopian tube carcinoma, recurrent ovarian carcinoma, recurrent primary peritoneal carcinoma	1	NCT03017131
	Cyclophosphamide	Lung cancer, non-small cell lung cancer		NCT03029273
	Pembrolizumab	Multiple myeloma	1	NCT03168438
NY-ESO-1 TCR + NY-ESO-1 peptide Vaccine	Aldesleukin, cyclophosphamide, fludarabine phosphate, nivolumab	Neoplasms	1	NCT02775292
NY-ESO-1 TCR+ NY-ESO-1 protein vaccine	Cyclophosphamide, fludarabine phosphate, aldesleukin, radiation	Solid tumors	1	NCT02366546
NY-ESO-1 protein vaccine	Atezolizumab, guadecitabine, carboxymethylcellulose, polyinosinic-polycytidylic acid, and poly-l-lysine (Poly ICLC)	Recurrent fallopian tube carcinoma Recurrent ovarian carcinoma Recurrent primary peritoneal carcinoma	1 and 2	NCT03206047
NY-ESO-1 peptide vaccine	Montanide ISA-51, polyinosinic-polycytidylic acid, and poly-l-lysine (Poly ICLC), Cyclophosphamide, Fludarabine, Interleukin-2	Melanoma	2	NCT02334735

Pre-clinical studies on plasmid DNA vaccine, encoding NY-ESO-1 epitopes, has shown induction of prophylactic anti-tumor cytotoxic T cell immune responses in vivo [83]. SCIB2, a DNA vaccine, encoding human IgG1 antibody, with 16 NY-ESO-1 epitopes (nested within the four regions of NY-ESO-1 covering the most common class I and class II haplotypes) has shown promising results in transgenic mice [84]. SCIB2 DNA constructs were found to induce CD4^+^/CD8^+^ T cell responses to NY-ESO-1 epitopes with long term survival in 35% of mice. Interestingly, enhanced antitumor responses with 100% long-term survival was observed when SCIB2 was used in combination with checkpoint inhibitors (anti-CTLA-4 and anti-PD-1 antibodies) [84]. Similarly, preclinical study on immunization of lung cancer mouse models with dendritic cell-targeting integration deficient based lentiviral vector), called LV305 demonstrated favorable results [85]. LV305, engineered to deliver NY-ESO-1 gene to human dendritic cells in vivo lead to presentation of NY-ESO-1 antigen peptides to naïve CD8^+^ T cells via MHC class I. Robust tumor specific cytotoxic T cell response against NY-ESO-1 expressing tumor with significant inhibition of metastatic lung nodule growth was reported in this study [85, 86]. Following the success of preclinical studies, recent phase 1 vaccination trial evaluating LV305 in NY-ESO-1 expressing solid tumors also showed promising results. LV305, was found to selectively induce NY-ESO-1 specific expression in dendritic cells and promoting tumor responses in patients [87]. Similarly, phase 1 study in synovial and myxoid round cell liposarcoma with CMB305 vaccine containing LV305 was shown to prime NY-ESO-1 specific T cell responses leading to enhanced overall survival [88].

Monoclonal antibodies (mAbs) against tumor-associated antigens have also shown promising results. Preclinical studies on combination therapy of NY-ESO-1 specific antibody, 12D7, with chemotherapy has shown to enhance efficacy of chemotherapy via local release of antigens by chemo- or radiotherapy. The released antigen forms an immune complex with mAbs, thus, allowing uptake and presentation of antigen-derived peptides by tumor associated dendritic cells leading to activation of tumor-specific CD8^+^ T cells [89]. Recently, new approaches, such as use of oncolytic viruses and antibody drug conjugates to target NY-ESO-1 have shown favorable results. For e.g. a study on human melanoma cell lines infected with oncolytic viruses (measles virus, vaccinia virus, vesicular stomatitis virus, herpes simplex type 1 virus, adenovirus or enterovirus) was able to stimulate anti-tumor immune response by inducing the release of NY-ESO-1. The released antigen captured by tumor cells and presented through HLA class 11 pathways leads to cytotoxicity activity and tumor lysis [90]. Furthermore, antibody drug conjugates, such as anti-CD-33 antibody drug conjugate, anti-CD30 antibody drug conjugates and anti-HER2 antibody conjugates have been shown to enhance antibody therapy against cancer cells. However, they have limited effect on intracellular protein. Therefore, novel TCR like antibodies have been developed that allow tumor specific antigens to go through MHC class 1 signaling to present it as tumor specific peptide/MHC complexes on tumor cell surface [91]. A study on NY-ESO-1 specific TCR like antibodies was shown to block recognition of NY-ESO-1/HLA-A2-positive tumour cells by NY-ESO-1peptide-specific CD8 + T cells thus enhancing tumour specific immunological response [92].

Another highly personalized therapeutic model involving NY-ESO-1 includes adoptive transfer of antigen-specific T cells (ACT) [93]. Clinical trials on adoptive transfer of T cells retro-virally transduced to express NY-ESO-1 T-cell repertoire (TCR) have been performed successfully for the treatment of melanoma and synovial sarcoma with response rates as high as 45% and 67% respectively. Furthermore, no on-target toxicities were observed in this trial indicating that ACT with TCRs directed against NY-ESO-1 is effective and safe at mediating tumor regression [94]. In addition to this, a clinical trial on multiple myeloma, utilizing ACT with TCRs directed against NY-ESO-1 showed encouraging results such as effective expansion of engineered T cells in vivo, efficient trafficking of T cells to disease site, long-term persistence/continued expression of TCR (for up to 2 years after infusion) and durable target-specific anti-tumor responses without significant safety concerns indicating clinical feasibility of NY-ESO-1 specific ACT for the management of myeloma [95]. Similarly, a pre-clinical study on glioblastoma, utilizing ACT with TCR directed against NY-ESO-1, in combination with Decitabine (DAC) chemotherapy has shown promising results [96]. Study results evidenced that prior administration of DAC to selectively up regulate NY-ESO-1 followed by ACT immunotherapy resulted in an efficient trafficking of NY-ESO-1-specific T cells towards tumor cells leading to survival advantage in mice [96]. Furthermore, phase I trial on chemo-immunotherapy, has been performed on relapsed/refractory solid tumors including Ewing’s sarcoma, osteosarcoma and rhabdomyosarcoma [97]. Chemo-immunotherapy phase I trial was based on combining DAC with a dendritic cell vaccine (DAC/DC-CT) targeting NY-ESO-1. DAC/DC-CT vaccine was well-tolerated, was able to elicit T cell responses in the majority of patients and achieved progression free survival in patients with minimum disease burden indicating the potential of chemo-immunotherapy in NY-ESO-1 expressing tumors [97].

Another well-known form of ACT, known as Chimeric Antigen Receptors (CARs) are constructed by linking variable regions of tumor-reactive antibody to intracellular signaling chains such as CD3-zeta, including costimulatory domains encoding CD28 or CD137 to fully activate T cells. The functional advantage of CARs is that it provides non-MHC–restricted recognition of cell surface components and can be introduced into T cells with high efficiency using viral vectors [93]. Pre-clinical data for the potential of CARs to be utilized for multiple myeloma has displayed encouraging results [98]. In this study, CARs recognizing the HLA-A*02:01/NY-ESO-1 peptide_157-165_ with intracellular moiety consisting of CD3z signaling domain (anti-NYESO-1-T1-CD3z) or a combined CD28/CD3z signaling domain (anti-NY-ESO-1-T1-CD28/CD3z) was generated. In cell lines, NY-ESO-1 specific cytolysis, IFNγ release, appearance of early and late effector T memory cells and differentiated effector T cells was observed. Furthermore, to observe the functional activity of anti-NY-ESO-1-T1-CD28/CD3z in vivo, a xenograft multiple myeloma mouse model was established. It was observed that mice were protected against tumor growth with measurable hIgE levels indicating the specificity of anti-NY-ESO-1-T1-CD28/CD3z T cells. In 40% of the blood samples, transduced T cells were present even after 30 days of treatment indicating a rationale for clinical trials against NY-ESO-1 positive myeloma cancer patients [98].

#### Improving therapeutic responses targeting NY-ESO-1

**Fig. 2:**
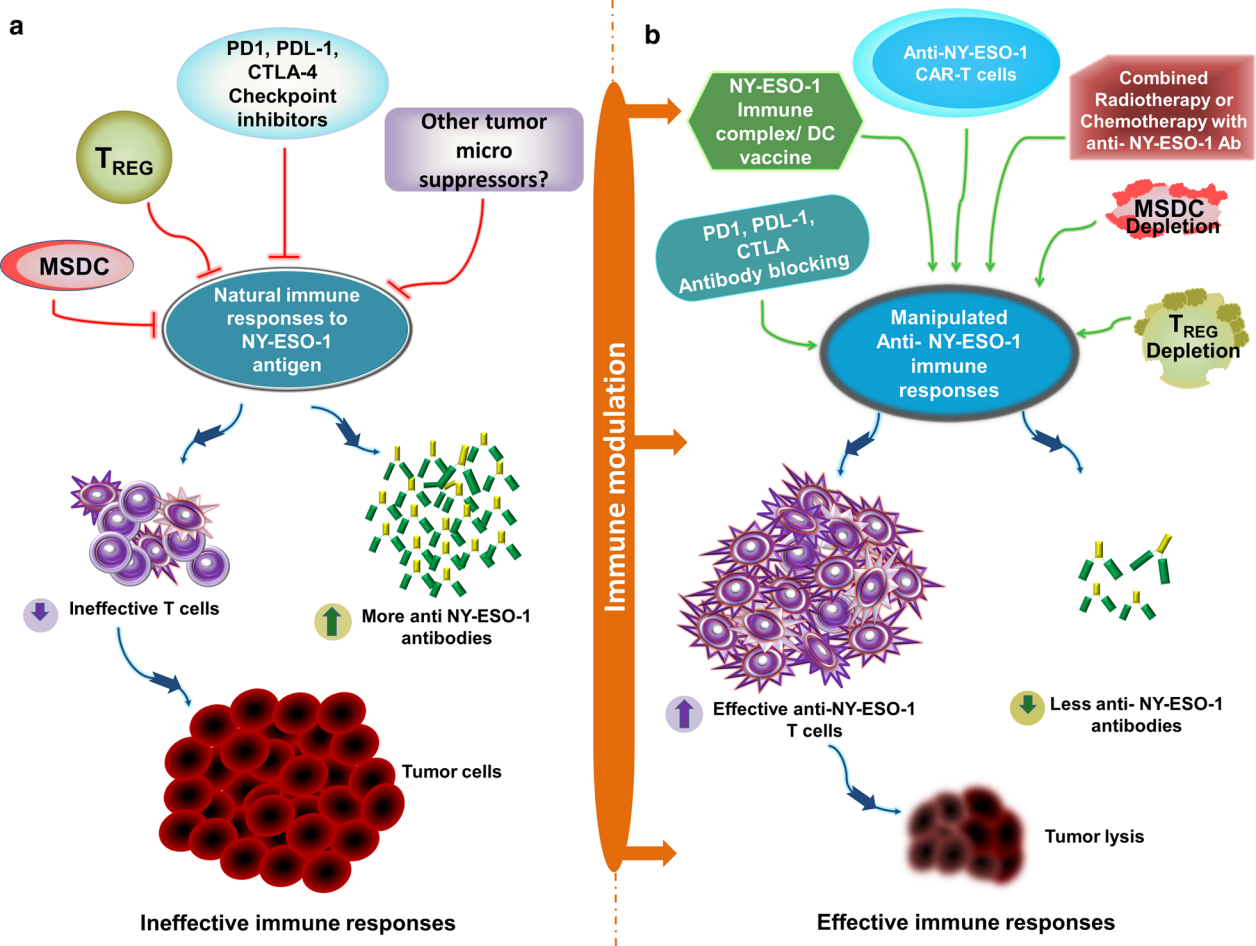
Cancer Immunotherapeutic strategies targeting NY-ESO-1 antigen. **a** NY-ESO-1 exhibits the capacity to induce a strong natural anti-NY-ESO-1 antibody, CD4 + and CD8 + T cell responses in an integrated manner. Effective tumor control against NY-ESO-1 expressing tumors is compromised due to strong interplay of the immune checkpoint inhibitory molecules such as programmed death 1 [PD-1], programmed death-ligand 1 [PDL-1], cytotoxic T-lymphocyte-associated protein 4 [CTLA-4] and other immune-suppressive tumor microenvironment cells such as regulatory T cells (TREG) and myeloid-derived suppressor cells (MDSC). In the presence of these immune inhibitory and immune suppressive cells, high titers of anti-NY-ESO-1 antibodies are observed while the anti-NY-ESO-1 T cell responses become ineffective. This leads to limited objective clinical responses to control tumors. **b** To strengthen the induction of effective anti-NY-ESO-1 specific CD4 + and CD8 + T cell immune responses and to reverse immunosuppression, various immune-modulation strategies including anti-PD-1, anti-PDL-1, anti-CTLA-4 blocking antibodies, TREG and MDSC depletion, NY-ESO-1 immune complex/dendritic cells (DC) vaccine, anti-NY-ESO-1 chimeric antigen receptor T cells (CAR T), either alone or in combination with standard of care therapies such as radiotherapy and chemotherapy can be designed for enhanced anti-NY-ESO-1 T cells responses leading to effective tumor eradication and a successful clinical response

NY-ESO-1 is a highly immunogenic antigen with a potential to induce integrated humoral and cellular responses. As described in Fig. 2a, effective tumor control against NY-ESO-1 expressing tumors is compromised due to strong interplay of the immune checkpoint inhibitory molecules such as programmed death 1 [PD-1], programmed death-ligand 1[PDL-1], cytotoxic T-lymphocyte-associated protein 4 [CTLA-4],immune-suppressive cells such as regulatory T cells (TREG) and myeloid-derived suppressor cells (MDSC). Various strategies to improve and strengthen NY-ESO-1 specific therapeutic responses including (1) vaccination with NY-ESO-1 protein/peptides complexed with dendritic cells can help to stimulate T cells with infiltration of cytotoxic cells; (2) adoptive T cell therapy with anti-NY-ESO-1 chimeric antigen receptor T cells (CAR T) facilitates T-cell expansion with favorable changes within the tumor microenvironment for tumor control; (3) Immune checkpoint inhibitors (anti-PD-1, anti-PDL-1, anti-CTLA-4) block inhibitory signals between T cells and NY-ESO-1 expressing tumor cells thus enhancing cellular responses and facilitating tumor control; (4) Depletion/blocking of immunosuppressive Tregs and MDSCs modulate the tumor microenvironment enhancing NY-ESO-1 specific cytotoxic responses; (5) Combining standard of care therapies, radiotherapy, or chemotherapy, with vaccination/adoptive T cell therapy/immune checkpoint inhibitors can enhance anti-NY-ESO-1 T cells responses leading to effective tumor eradication and a successful clinical response (Fig. 2b).

## Conclusion

Comprehensive and compelling data from preclinical/clinical trials on NY-ESO-1 evidence its dynamic potential as a candidate for immunotherapy for a variety of malignancies. However, further trials and pre-clinical data are in the pipeline that will help to decipher its inherent and powerful immunological prowess to its full potential.

## Future perspectives

The future of immunotherapy relies largely on selected tumor antigens such as NY-ESO-1 due to its natural ability to induce both humoral and cellular immune responses. The major aim of researchers now is to counter potential roadblocks in therapeutic pathways encompassing NY-ESO-1. This can potentially be achieved via combination therapy to increase the efficacy of NY-ESO-1 specific immunotherapeutic interventions including (a) vaccines in combination with Tregs depletion and/or blocking of immune checkpoint inhibitors; (b) adoptive T cell therapy with vaccination to boost T cell responses post adoptive transfer; (c) adoptive T cell therapy with immune checkpoint inhibitors/Tregs depletion; (d) antibodies in combination with vaccination/adoptive transfer; (e) NY-ESO-1 vaccine in combination with other cancer testis antigens and f) vaccination/ACT/antibodies in combination with standard of care therapies such as radiation, chemo-radiation and surgery. These strategies will not only enhance the immunogenic potential of NY-ESO-1 but will also be beneficial in overcoming resistance to therapeutic interventions.

## Data Availability

Not applicable.

## Abbreviations

NY-ESO-1: New York esophageal squamous cell carcinoma 1
CTA: Cancer testis antigen
TAA: Tumor-associated antigens
TCR: T cell repertoire
CT6: Cancer testis 6 antigen
CSC: Cancer stem cells
MSC: Mesenchymal stem cells
RT-PCR: Reverse transcriptase PCR
IHC: Immunohistochemistry
APC: Antigen presenting cells
Tregs: Regulatory T cells
MDSC: Myeloid-derived suppressor cells
ISCOMATRIX: Saponin based adjuvant
CHP: Cholesteryl Pullulan
Poly-ICLC: Polyinosinic-polycytidylic acid-poly-l-lysine carboxymethylcellulose
MAbs: Monoclonal antibodies
ACT: Antigen-specific T cells
DAC: Decitabine
PD-1: Programmed cell death protein 1
PD-L1: Programmed death-ligand 1
CTLA-4: Cytotoxic T-lymphocyte-associated protein 4
CAR: Chimeric antigen receptor
